# Supplementary material for: Revealing solid electrolyte interphase formation through interface-sensitive Operando X-ray absorption spectroscopy
Source: Nat Commun. 2022 Oct 14;13:6070. doi: 10.1038/s41467-022-33691-1 (PMC9568580; doi:10.1038/s41467-022-33691-1)
Supplement: Supplementary file 1 — Supplementary Information [file 41467_2022_33691_MOESM1_ESM.pdf]

Revealing Solid Electrolyte Interphase Formation Through Interface-Sensitive *Operando* X-ray Absorption Spectroscopy - Supplementary Information

Jack E. N. Swallow[1,2,3], Michael W. Fraser[1,3], Nis-Julian H. Kneusels[4], Jodie F. Charlton[1,2], Christopher G. Sole[2,3], Conor M. E. Phelan[1], Erik Björklund[1,3], Peter Bencok[2], Carlos Escudero[5], Virginia Pérez-Dieste[5], Clare P. Grey[4], Rebecca J. Nicholls[1] and Robert S. Weatherup[1,2,3*]

[1]Department of Materials, University of Oxford, Parks Road,Oxford, OX1 3PH, United Kingdom.
[2]Diamond Light Source, Didcot, Oxfordshire, OX11 0DE, United Kingdom.
[3]The Faraday Institution, Quad One, Harwell Science and Innovation Campus, Didcot, OX11 0RA, United Kingdom.
[4]Department of Chemistry, University of Cambridge, Lensfield Road, Cambridge, CB2 1EW, United Kingdom.
[5]ALBA Synchrotron Light Source, Carrer de la Llum 2-26, 08290 Cerdanyola del Vallès, Barcelona, Spain.
[*]robert.weatherup@materials.ox.ac.uk.

Operando Cell Electrochemsitry:

Figure S1 shows the voltage profiles of a-Si(100 nm) cycled using the *operando* cells set-up (described in the main manuscript) in a half cell configuration with LP30 electrolyte. Potential cutoffs were set at 100 mV (pink) and 5 mV (purple). A similar behaviour is seen to that of the coin cells in Figure 1b of the main manuscript. We note that the active material mass used for the *operando* cell is based on the areal density of the a-Si electrode and the area in contact with the electrolyte as defined by a viton O-ring. The capacity at the 100 mV cutoff potential is slightly lower than that of the coin cells cycled to the same potential in Figure 1b which we attribute to a large uncertainty in the electrode area defined by the viton O-ring, depending on the level of compression. The *operando* cells were discharged/charged at room temperature at a constant current (C/30, 120 mA/g) for the first cycle using a Biologic SP-300 potentiostat, and then cycled at C/10 (360 mA/g) for up to 10 cycles.

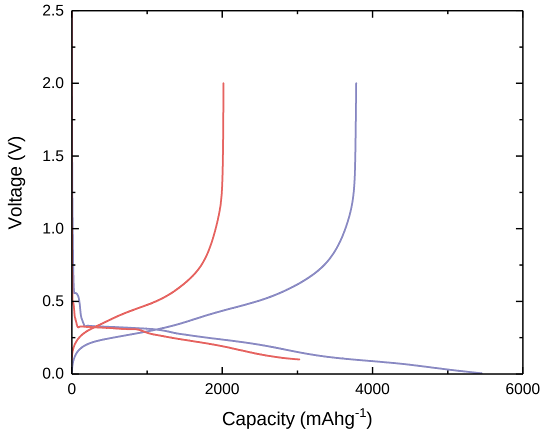

Fig. S1 Voltage profiles using *operando* cell. Voltage profiles using the *operando* cell for the first cycle containing LP30 electrolyte with no additive, cycled to 100 mV (pink) and 5 mV (purple).

Cycled Si K-edge:

Figure S2 shows Si K-edge TEY-XAS measurements of the as-deposited a-Si(20 nm)/Ni(20 m) electrodes acquired *ex situ*, and at at a variety of potential in the *operando* cell filled with LP30 electrolyte. (i) As-deposited electrode, which displays spectral features matching both Si (peak at ∼1842 eV) and SiO_2 (peak at ∼1848 eV). Formation of a native SiO_2 layer is expected as the material is transported through air. (ii) Electrodes cycled to 0.6 V in LP30, the spectrum closely resembles that of elemental Si, indicating the native SiO_2 has been electrochemically reduced. (iii) Electrodes cycled to 0.2 V and 0.1 V, a strong peak at ∼ 1846 eV emerges which we assign to Li-silicide,[1] demonstrating lithiation of the Si in correspondence with Figure 1c of the main manuscript. Schematics illustrating the proposed detection mechanism involving core-hole creation, electron emission, subsequent electron replenishment are shown in the right hand panel for (i-iii).

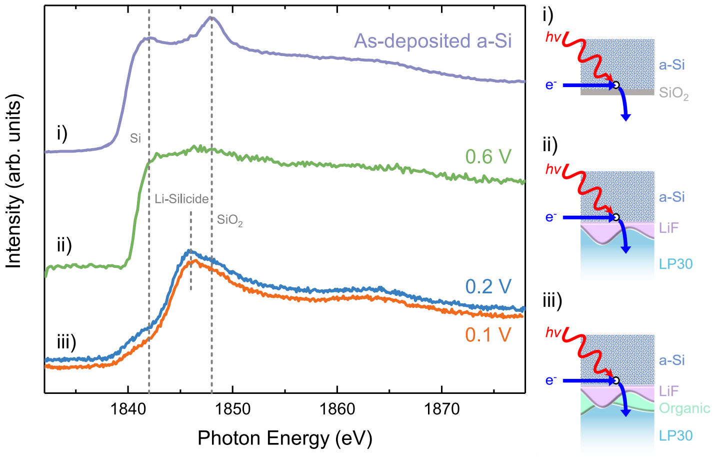

Fig. S2 ***Operando* TEY-XAS of Si K-edge.** Si K-edge TEY-XAS of as-deposited a-Si(20 nm)/Ni(20 m) on Si_3N_4(100 m) membranes measured *ex situ* and in the *operando* cell containing LP30 at different potentials ranging from 0.6 V down to 0.1 V. Schematic representations of the different electron emission and replenishment paths following photoexcitation is shown for the different measurements, with labels (i-iii) corresponding to the spectra. The energies of the main features for Si (1842 eV), SiO_2 (1848 eV), and Li_xSi (1846 eV) are indicated.[1–3].

Si Reference Measurements:

Figure S3 shows the O and Si K-edges for as-deposited a-Si(600 nm), Ni(20 nm) deposited onto Si_3N_4(100 nm), and a-Si(20 nm)/Ni(20 nm) deposited onto Si_3N_4(100 nm). The spectrum for SiO_2 is clearly seen in the O K-edge in each case, with the addition of a peak at 532 eV associated with oxidised Ni for the Ni(20 nm) deposited onto Si_3N_4 sample. This NiO peak is no longer seen when a-Si(20 nm) is deposited on top, consistent with the NiO being buried at a depth greater than the ∼10 nm range of electrons detected by TEY-XAS of the O K-edge. It may also be the case that the thin NiO layer is sputtered away to some extent by the energetic Si atoms impinging during sputter-deposition of the a-Si layer.

In the Si K-edge data, both the a-Si(600 nm) and a-Si(20 nm)/Ni(20 nm)/Si_3N_4 samples display signals related to Si and SiO_2. The SiO_2 is attributable to native oxide formation with the relative intensities varying due to the different thicknesses of a-Si, with greater Si contribution from the a-Si(600 nm). For the Ni(20 nm)/Si_3N_4 sample Si_3N_4 (1486.5 eV) and SiO_2 are clearly visible, and are expected to arise from the buried Ni/Si_3N_4 interface. This is consistent with the greater range of Auger electrons at the higher energy Si K-edge (∼30 nm) compared to the O and F K-edges (∼10 nm).

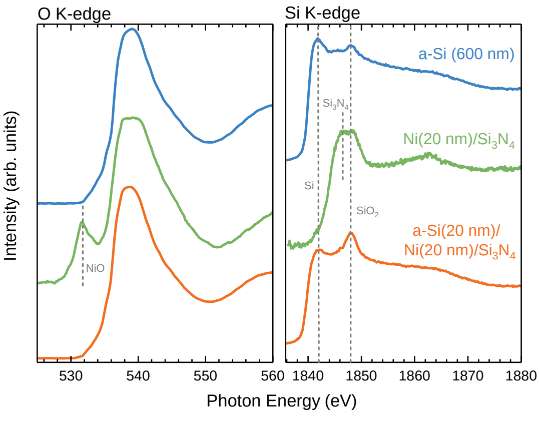

Fig. S3 O K-edge and Si K-edge spectra of reference Si materials. TEY-XAS of the O K-edge and Si K-edge for as-deposited a-Si(600 nm)/Ni(250 nm) on Cu foil, Ni(20 nm) on Si_3N_4(100 nm), and a-Si(20nm)/Ni(20 nm) on Si_3N_4(100 nm). The line shapes of the O K-edges resemble that of oxidised Si, with a pre-edge in the case of Si_3N_4+Ni(20nm) due to NiO being present. The line shapes of the Si K-edges vary significantly, and the energies of the main features for Si (1842 eV) and SiO_2 (1848 eV), and Si_3N_4 (1486.5 eV) are indicated.[1, 2] Whilst Si and SiO_2 are both present in the a-Si(600 nm) and a-Si(20 nm)/Ni(20nm)/Si_3N_4 samples, the ratios of the two peaks vary, demonstrating that we measure a higher proportion of SiO_2 relative to Si in the thin film rather than the bulk Si. The presence of Si_3N_4 features for Ni(20nm)/Si_3N_4, but not a-Si(20 nm)/Ni(20nm)/Si_3N_4 indicates a probing depth of >20 nm but <40 nm. Additionally, whilst Si_3N_4 and Li_xSi have features at similar energies (see Figure S2), their spectral shapes vary sufficiently to give confidence in their assignment here.[2, 3]

LiF Reference Spectrum:

Figure S4 shows TEY-XAS for LiF powder compared to the LiF spectrum recorded at 0.1 V in the *operando* cell. The sharper features seen in the reference spectrum compared to the spectrum from the *operando* cell, are attributable to the reference measurement having been acquired on a different beamline (B07 at Diamond Light Source), using beam parameters that give higher resolution. Other than this the spectral features are replicated very well. Additionally, the peak attributed to excitonic effects in the main manuscript (~690.9 eV) is more clearly visible in the reference spectrum.

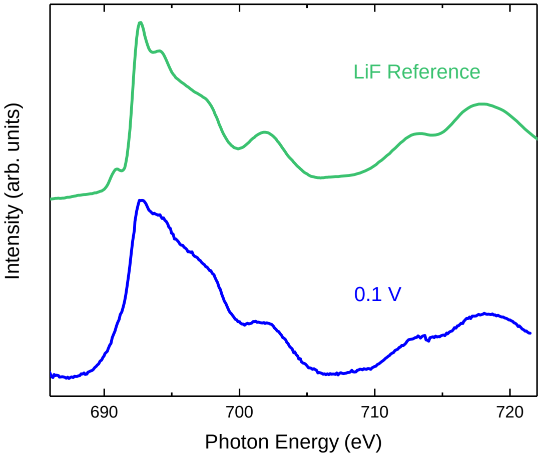

Fig. S4 F K-edge spectra for LiF powder and a-Si cell at low potential. TEY-XAS of the F K-edge for a powder LiF reference compared to the LiF spectrum measured at a hold potential of 0.1 V in the *operando* cell. The *operando* data is broader, but matches the spectral features of the reference extremely well. The peak attributed to excitonic effects in the main manuscript (~690.9 eV) is more clearly visible in the reference spectrum.

DFT Geometry Optimisation:

Table S1 gives a list of the converged parameters used in the geometry optimisation calculations performed with the CASTEP code, including energy cutoff and k-point grid sizes for the material systems studied here. The geometry of the system was considered optimized when the maximum forces on the ions was below 0.01 eV/Å. We also include the calculated bond lengths for the optimised geometries of each material system studied, and provided an experimental comparison where one could be found.

Material	Energy cutoff (eV)	k-point spacing (grid)	Bond	Length DFT (Å)	Length Experimental (Å)	
EC (Mol.)	800	0.03 ($4 \times 4 \times 4$)			Soetens et al.[4]	Matias et al.[5]
			O1-C1	1.1991	1.20	1.2016
			C1-O2	1.3681	1.34	1.3404
			O2-C2	1.4427	1.46	1.4556
			C2-C3	1.5274	1.52	1.5190
			C2-H1	1.1001	-	1.0936
			C3-H3	1.0954	-	1.0893
DMC (Mol.)	800	0.03 ($3 \times 3 \times 4$)			Mulhoff[6]	
			O1-C1	1.2149	1.203	
			C1-O2	1.3474	1.343	
			O2-C2	1.4398	1.423	
			C2-H1	1.0941	1.100	
			C2-H2	1.0975	1.100	
LiF (Crys.)	800	0.015 ($29 \times 29 \times 29$)			Harrison[7]	
			L-F	2.0318	2.014	
$LiPF_6$ (Crys.)	800	0.03 ($9 \times 9 \times 9$)				
			P-F	2.0785	-	
PF_6^- (Mol.)	800	0.03 ($4 \times 4 \times 4$)				
			P-F	1.6434	-	

Table S1 Parameters used in DFT geometry optimisation of molecules and crystals. A comparison of the converged parameters used in the geometry optimisation DFT calculations (see Figure S5 for atom labels of the EC and DMC molecules). Experimentally determined bond lengths are also shown for comparison.

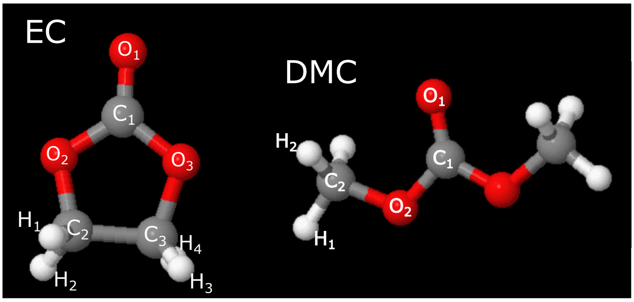

Fig. S5 Atomic designations for the EC and DMC molecules. Geometry optimised ethylene carbonate and dimethyl carbonate molecules. The atom designations match those in table S1.

Isosurfaces:

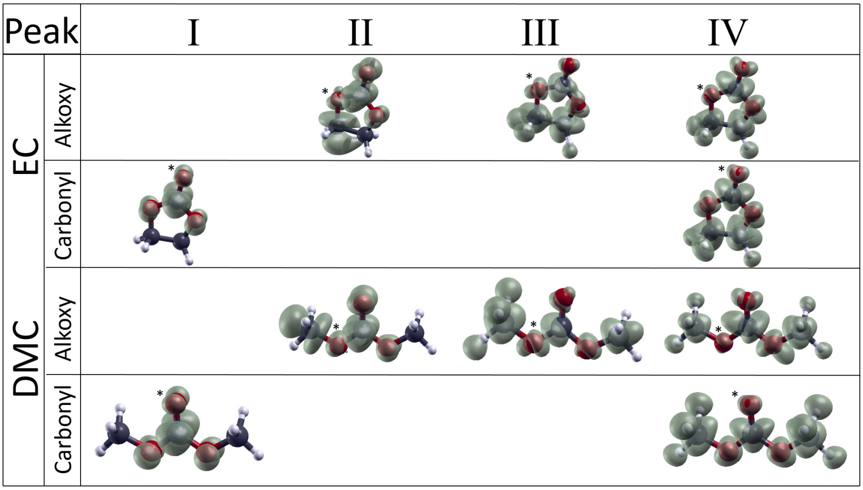

Fig. S6 DFT calculated isosurfaces for EC and DMC molecules. Isosurface of the excited states seen in Figure 4a of the main manuscript, formed from the bands which contribute to the peaks I-IV in the O p-DOS (isosurface value 0.5 eÅ^{-3} for all but the bands in peak III, where 1.1 eÅ^{-3} was used due to the higher number of bands). An asterisk is used to identify which oxygen atom the core-hole was placed on.

Figure S6 displays isosurfaces projected around the atoms, which have been used here to help assign the spectral features seen in Figure 4a of the main manuscript. We have used the c2x program to prepare the CASTEP output into a format for visualisation[8], while visualisation of the isosurfaces is performed with the XCrySDen software[9]. Two core-hole spectra were generated per molecule, a core-hole place on the carbonyl oxygen and one on an alkoxy oxygen atom (the two alkoxy oxygen atoms are inequivalent). The positioning of the core-hole is reflected in the column titles in Figure S6 in each case, and the oxygen atom onto which the core-hole is placed is identified with an asterisk in Figure S6. Visualisation is performed by selecting the appropriate bands comprising the four features (peaks I-IV in Figure 4a) in the X-ray absorption spectrum, and setting an isosurface value which aides in visualisation of the density (isosurface values of 0.5 eÅ^{-3} were used for all but the bands in peak III, where 1.1 eÅ^{-3} was used due to the higher number of bands). Note that unlike in Figure 4a in the main manuscript, the orbitals projected here represent the total density of states, and so are not specific to the core-hole transition, although this is included in the projection.

We see the sharp peaks I and II at low energy derive from the EC/DMC carbonyl and alkoxy oxygens respectively, and represent π^* antibonding orbitals, as discerned from their highly localized nature and the alignment of the orbitals around the carbon and oxygen atoms being projected perpendicular from the

bond direction. The density around the molecule when the core-hole is place on the alkoxy oxygen atom is asymmetric, with a smaller, more contracted electron density on the core-hole atom itself due to a higher positive potential, and greater density on the carbon atom directly below (in EC) or on the left of (in DMC) the core-hole oxygen. This again is related to charge redistribution due to the effect of the removal of an electron and is consistent across all the projection for peak II-IV, apart from that of the DMC in peak III where the projected DOS around the core-hole oxygen is influenced by the adjacent carbon giving slightly larger density. Peak III and IV display far more dispersive character due to the greater density of bands over these energies which makes it more difficult to identify the exact nature of the bonds involved, but importantly the orbitals from the O-C bonds are directed along the bond direction, indicative of σ^* type bonding. There is no strong evidence in Figure S6 as to why the alkoxy core-hole oxygen atoms in EC display a clear peak IV while where is non for the DMC, although the projected density is noticeably smaller in the around the core-hole oxygen of the DMC which may give some explanation.

DFT Core-Hole Spectral Calculations:

Material	Energy cutoff (eV)	k-point spacing (grid)	Spectral k-point spacing (grid)	Cell size a-length (Å)
EC (Mol.)	800	0.03 (4×4×4)	0.01 (9×9×9)	12
DMC (Mol.)	800	0.03 (3×3×4)	0.01 (9×9 ×9)	12
LiF(Crys.)	800	0.03 (5×5×5)	0.01 (15×15×15)	8.62
$LiPF_6$(Crys.)	800	0.03 (9×9×9)	0.01 (12×12×12)	10.38
PF_6^- (Mol.)	800	0.03 (4×4×4)	0.01 (11×11×11)	10

Table S2 Parameters used in DFT core-hole calculations of molecules and crystals. A comparison of the parameters used for the spectral (core-hole) calculation.

Table S2 shows the converged parameters used in all of the core-hole spectral calculations performed. The treatment of the materials is defined as either molecular (Mol.) or crystalline (Crys.) which indicates either a molecule in a box or an infinite crystal description. Convergence here was determined when a negligible change between the generated spectra was observed upon changing the calculation parameters (to higher energy cutoff and lower k-point spacing/larger k-point grid used to generate the density and the spectrum). We also provide the cell sizes used in the calculation which were also tested for convergence in the same manner. Since the cells were all cubic, only the a-length parameter is given.

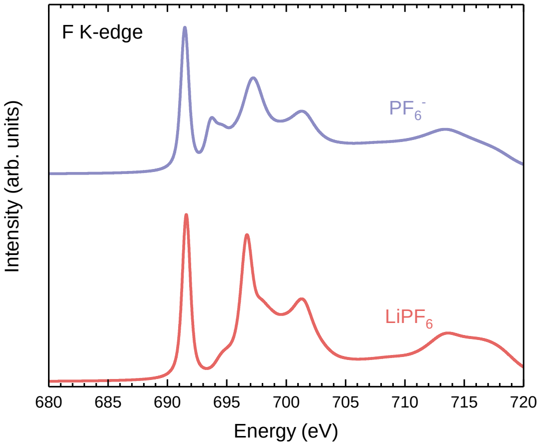

Fig. S7 DFT calculated F K-edge spectra for PF_6^- and $LiPF_6$. Core-hole calculated F K-edge spectra of PF_6^- and $LiPF_6$, each aligned to the first peak of the experimental data. We apply a 0.5 eV width Gaussian and an energy dependent 0.5 eV width Lorentzian broadening to the data sets here to aid comparison with experimental data.

Figure S7 shows the DFT calculated core-hole spectra for both a PF_6^- molecule in a box, and an $LiPF_6$ infinite crystal (see tables S1 and S2 for more information on the calculations performed). The spectra have been aligned to

the first peak of the experimental data and we have applied a 0.5 eV width Gaussian and a 0.5 eV width Lorentzian broadening (which we make energy dependent using the formula $\Gamma_L = \Gamma_{L(0.5)} + 0.1(E - E_f)$ to account for the energy dependence of the excited state lifetime) to the data sets. As expected, the spectral features are similar between the $PF_6{}^-$ and $LiPF_6$ systems, both displaying peaks representative of the octahedral coordination they share. The peak intensities and widths vary slightly, with the $PF_6{}^-$ having a relatively more intense peak at ~694 eV, broader peaks at energies above the first absorption edge (>694 eV). This broadening seen for the $PF_6{}^-$ ions may contribute to the very broad peaks seen in the experimental data in Figures 3b and 6b of the main manuscript.

Comparison of Experimental and Theoretical Valence Density of States:

Figure S8 shows the experimentally determined valence total density of states (DOS) as measured via XPS by Dedryvére et al.[10] and the theoretically determined partial density of states (pDOS) as determined from our simulations. The optimised $LiPF_6$ geometry was implemented for the pDOS calculation, and the energy cutoff was set to 800 eV and the k-point grid to $24 \times 24 \times 24$. We did not place a core-hole on any of the atoms for the DOS calculation. The simulated spectrum was broadened using a 0.6 eV width Gaussian (matching the width of a Ag $3d_{5/2}$ core level also measured by Dedryvére et al.[10]) and 0.9 eV width energy dependent Lorentzian. The simulated spectrum is seen to match the data extremely well, with the valence peaks aligning in energy and intensity. The semi-core F 2s is slightly lower in energy for the simulated spectrum, which us attributable to the PBE functional used.

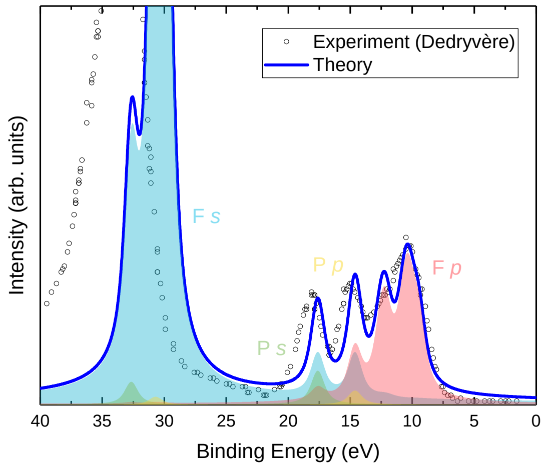

Fig. S8 DFT calculated density of states for $LiPF_6$. Theoretically determined total density of states and valence band XPS from Dedryvére et al.[10] of $LiPF_6$ salt. Spectral broadening has been applied to the theory using a 0.6 eV width Gaussian and 0.9 eV width energy dependent Lorentzian, matching the data reasonably well. This close alignment gives increased confidence in the theoretical description of the $LiPF_6$ structure.

Excitation Energies of the O $1s \rightarrow \pi^*$ Transitions:

Table S3 presents a large number of experimentally determined O $1s \rightarrow \pi^*$ transition energies taken from the O K-edge XAS data from various sources. This data is used in Figure 5b of the main manuscript.

Molecule	Formula	O K-edge excitation energy (eV)	Ref.
Dimethyl carbonate	$OC(OCH_3)_2$	532.93	11
Sodium carbonate	Na_2CO_3	534.0	12
Sodium bicarbonate	$NaHCO_3$	534.0	12
Barrium carbonate	$BaCO_3$	533.2	13
Lithium carbonate	Li_2CO_3	533.9	14
Propylene carbonate	$C_4H_6O_3$	533.3, 533.5	15, 16
Lithium ethylene dicarbonate	$(CH_2OCO_2Li)_2$	533.7	17
Ethylene carbonate + dimethyl carbonate	EC+DMC	533.3	15
Ethyl benzoate	$C_9H_{10}O_2$	531.5	18
Dimethyl terephthalate	$C_6H_4(COOCH_3)_2$	531.5	19
Acetic acid	CH_3COOH	532.0, 532.1, 532.3	20–22
Lithium acetate	$C_2H_3LiO_2$	532.4	17
Formic acid	H_2CO_2	532.2	23
Zinc acetate	$Zn(CH_3CO_2)_2$	532.8	24
Oxalic acid	$C_2H_2O_4$	531.7	25
Acetone	C_3H_6O	531.3,531.4,531.5,531.3	22, 23, 26, 27
Acetaldehyde	C_2H_4O	531.1,531.5	23, 26
Benzaldehyde	C_7H_6O	531.0	18
Terephthaldehyde	$C_8H_6O_2$	530.6	18
Lithium oxide	Li_2O	534.3	14
Polyethylene terephthalate	$(C_{10}H_8O_4)n$	531.2	28
Polybutylene terephthalate	$(C_{12}H_{12}O_4)n$	531.2	28
4,4'-Biphenyldicarboxylic Acid Dimethyl Ester	$C_{16}H_{14}O_4$	531.1	28
4,4'-Biphenyl dicarboxylic Acid	$C_{14}H_{10}O_4$	531.7	28
Terephthalic Acid	$C_8H_6O_4$	532.4	28

Table S3 Molecular O K-edge excitation energies for O=C related molecules. Excitation energies of the O $1s \rightarrow \pi^*$transition for a number of molecules possessing an O=C bonding configuration and either two, one or no O-C bonds. This data is presented graphically in Figure 5b of the main manuscript.

O K-edge Without Bias and TEY of Reference Materials:

Figure S9 displays FY-XAS of the O K-edge taken when the cell is held at 0.05 V, overlaid with a spectrum taken when the potential is completely removed after ∼ 30 mins of relaxation. the spectral features remain unchanged, effectively demonstrating that the bias has no effect on the XA spectra measured. Also shown in Figure S9 are the TEY-XA spectra of the reference powders: lithium acetate, lithium oxalate and lithium formate, which nicely demonstrate the energy positions of related features in comparison to the spectra measured from our cell. The TEY data is expected to have slightly better resolution that the TFY, although it is more subject to surface oxides which may account for some of the features.

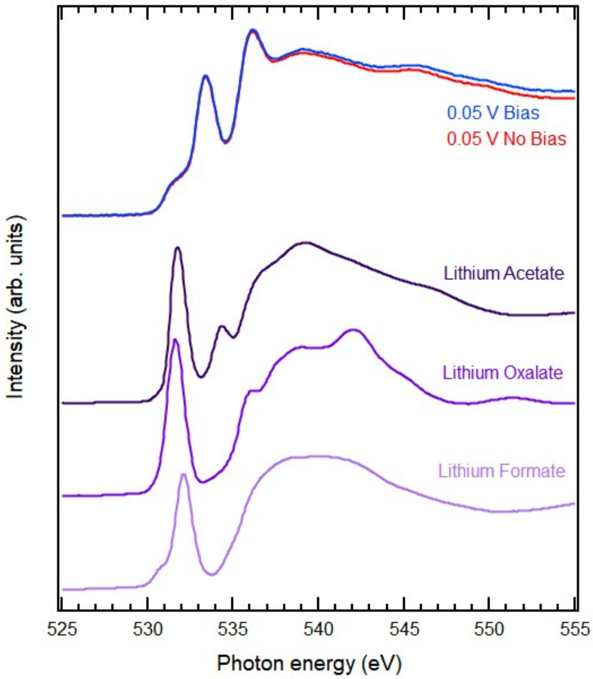

Fig. S9 O K-edge spectra of *operando* cell and reference powders. Top: FY-XAS of the O K-edge taken when the cell is held at 0.05 V, and then again when the potential is completely removed. Bottom: TEY-XAS of reference powders.

O K-edge Quick Scans:

Figure S10 displays rapid TEY-XAS scans of the regions of the O K-edge corresponding to the O 1s $\rightarrow$ π^* transitions for carbonate species. The data was recorded in the *operando* cell using the LP30 electrolyte (Figure S10a), and LP30+FEC additive (Figure S10b). Notice how the peak only begins to reduce in intensity when a hold potential of 0.4 V is applied when only LP30 is used, but this threshold is increased to 1.0 V when the FEC additive is present. The reduction of the peak intensities occurs over a period of ~10-15 mins in both cases.

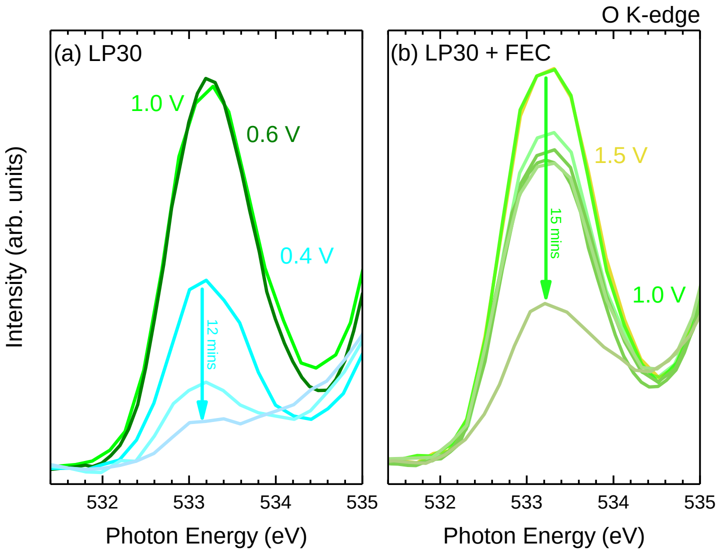

Fig. S10 Rapid O K-edge scans of a-Si cell with and without FEC additive. TEY-XAS of the O K-edge region (peak I from Figure 3-6 of the main manuscript) taken using the *operando* cell with (a) LP30 electrolyte and (b) LP30+FEC additive. In (a), spectra are acquired at a hold potential of 1.0 V before decreasing the potential to 0.6 V and finally 0.4 V. No change is visible between the two spectra at 1.0 V and 0.6 V, it is only when 0.4 V is applied that the peak clearly reduces in intensity, with the changes seen occurring over a period of 12 mins. In (b) spectra are acquired at a hold potential of 1.5 V before decreasing the potential to 1.0 V. A sizable change is seen at 1.0 V over a period of 15 mins, where the peak intensity is seen to decrease. This demonstrates firstly the time period over which these changes occur, and secondly that these changes happen at a higher potential when FEC is present as an additive in the LP30 electrolyte.

Raman Spectroscopy:

Figure S11 shows the Raman spectra of our deposited amorphous Si (a-Si, 100 nm) thin film electrode material, which is deposited via RF magnetron sputtering onto Ni (250 nm) on Cu foil, and a reference crystalline Si (c-Si) wafer. The Raman modes match those seen in the literature for both a-Si and c-Si respectively[29], giving confidence in the fully amorphous nature of the as deposited Si.

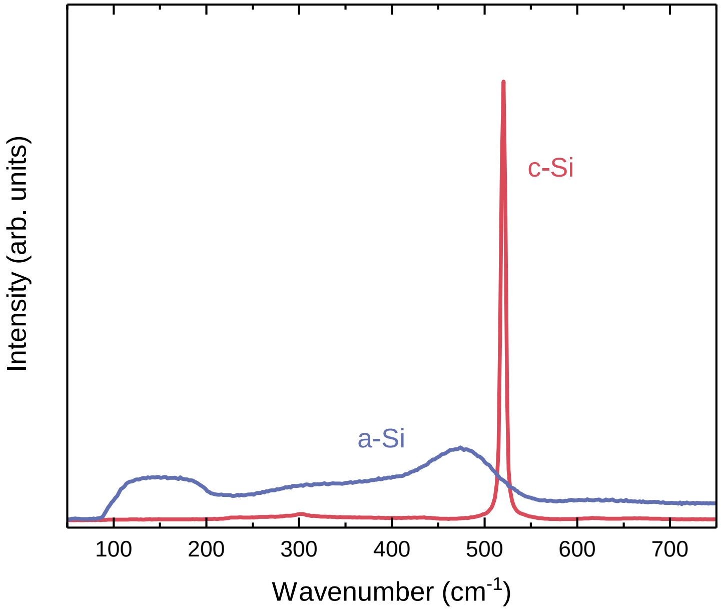

Fig. S11 Raman spectroscopy of a-Si electrode and c-Si wafer. Raman spectra of as deposited a-Si (100 nm) thin film on Ni (250 nm) on Cu foil, and reference c-Si wafer.

References

[1] Yamada, M., Inaba, A., Ueda, A., Matsumoto, K., Iwasaki, T., Ohzuku, T.: Reaction mechanism of "SiO"-carbon composite-negative electrode for high-capacity lithium-ion batteries. J. Electrochem. Soc. **159**(10), 1630–1635 (2012). https://doi.org/10.1149/2.018210jes

[2] Wilson, P.R., Roschuk, T., Dunn, K., Normand, E.N., Chelomentsev, E., Zalloum, O.H., Wojcik, J., Mascher, P.: Effect of thermal treatment on the growth, structure and luminescence of nitride-passivated silicon nanoclusters. Nanoscale Res. Lett. **6**, 168 (2011). https://doi.org/10.1186/1556-276X-6-168

[3] Wang, B., Liu, J., Norouzi Banis, M., Sun, Q., Zhao, Y., Li, R., Sham, T.-K., Sun, X.: Atomic layer deposited lithium silicates as solid-state electrolytes for all-solid-state batteries. ACS Appl. Mater. Interfaces **9**(37), 31786–31793 (2017). https://doi.org/10.1021/acsami.7b07113. PMID: 28749129

[4] Soetens, J.-C., Millot, C., Maigret, B., Bakó, I.: Molecular dynamics simulation and x-ray diffraction studies of ethylene carbonate, propylene carbonate and dimethyl carbonate in liquid phase. J. Mol. Liq. **92**(3), 201–216 (2001). https://doi.org/10.1016/S0167-7322(01)00192-1

[5] Matias, P.M., Jeffrey, G.A., Wingert, L.M., Ruble, J.R.: Single crystal neutron diffraction analysis (15 k) and ab initio molecular orbital calculations for ethylene carbonate. J. Mol. Struct: THEOCHEM **184**(3), 247–260 (1989). https://doi.org/10.1016/0166-1280(89)85094-8

[6] Mulhoff, F.C.: The molecular structure of dimethylcarbonate, determined by gas electron diffraction. J. Mol. Struct. **36**(2), 334–335 (1977). https://doi.org/10.1016/0022-2860(77)85067-9

[7] Harrison, W.A.: Electronic Structures and the Properties of Solids: The Physics of the Chemical Bond. Dover Publications Inc (1989)

[8] Rutter, M.J.: C2x: A tool for visualisation and input preparation for castep and other electronic structure codes. Comput. Phys. Commun. **225**, 174–179 (2018). https://doi.org/10.1016/j.cpc.2017.12.008

[9] Kokalj, A.: Xcrysden—a new program for displaying crystalline structures and electron densities. J. Mol. Graph. Model. **17**(3), 176–179 (1999). https://doi.org/10.1016/S1093-3263(99)00028-5

[10] Dedryvére, R., Leroy, S., Martinez, H., Blanchard, F., Lemordant, D., Gonbeau, D.: XPS valence characterization of lithium salts as a tool to study electrode/electrolyte interfaces of Li-ion batteries. J. Phys. Chem.

B **110**, 12986–12992 (2006). https://doi.org/10.1021/jp061624f

[11] Urquhart, S.G., Ade, H.: Trends in the carbonyl core (C 1s, O 1s) $\rightarrow$ $\pi^*_{C=O}$ transition in the near-edge x-ray absorption fine structure spectra of organic molecules. J. Phys. Chem. B **106**(34), 8531–8538 (2002). https://doi.org/10.1021/jp0255379

[12] Espinal, L., Green, M.L., Fischer, D.A., DeLongchamp, D.M., Jaye, C., Horn, J.C., Sakwa-Novak, M.A., Chaikittisilp, W., Brunelli, N.A., Jones, C.W.: Interrogating the carbon and oxygen k-edge nexafs of a CO_2-dosed hyperbranched aminosilica. J. Phys. Chem. Lett. **6**(1), 148–152 (2015). https://doi.org/10.1021/jz502483v

[13] Karslıoğlu, O., Trotochaud, L., Zegkinoglou, I., Bluhm, H.: X-ray spectroscopic characterization of BaO, $Ba(OH)_2$, $BaCO_3$, and $Ba(NO_3)_2$. J. Electron Spectros. Relat. Phenomena **225**, 55–61 (2018). https://doi.org/10.1016/j.elspec.2018.03.008

[14] Qiao, R., Chuang, Y.-D., Yan, S., Yang, W.: Soft x-ray irradiation effects of Li_2O_2, Li_2CO_3 and Li_2O revealed by absorption spectroscopy. PLoS ONE **7**, 49182 (2012). https://doi.org/10.1371/journal.pone.0049182

[15] Ketenoglu, D., Spiekermann, G., Harder, M., Oz, E., Koz, C., Yagci, M.C., Yilmaz, E., Yin, Z., Sahle, C.J., Detlefs, B., Yavaş, H.: X-ray raman spectroscopy of lithium-ion battery electrolyte solutions in a flow cell. J. Synchrotron Rad. **25**, 537–542 (2018). https://doi.org/10.1107/S1600577518001662

[16] Smith, J.W., Lam, R.K., Sheardy, A.T., Shih, O., Rizzuto, A.M., Borodin, O., Harris, S.J., Prendergast, D., Saykally, R.J.: X-ray absorption spectroscopy of $LiBF_4$ in propylene carbonate: a model lithium ion battery electrolyte. Phys. Chem. Chem. Phys. **16**, 23568–23575 (2014). https://doi.org/10.1039/c4cp03240c

[17] Zhuo, Z., Lu, P., Delacourt, C., Qiao, R., Xu, K., Pan, F., Harris, S.J., Yang, W.: Breathing and oscillating growth of solidelectrolyte-interphase upon electrochemical cycling. Chem. Commun. **54**, 814 (2018). https://doi.org/10.1039/c7cc07082a

[18] Hitchcock, A.P., Urquhart, S.G., Rightor, E.G.: Inner-shell spectroscopy of benzaldehyde, terephthalaldehyde, ethylbenzoate, terephthaloyl chloride and phosgene: models for core excitation of poly(ethylene terephthalate). J. Phys. Chem. **96**(22), 8736–8750 (1992). https://doi.org/10.1021/j100201a015

[19] Urquhart, S.G., Hitchcock, A.P., Smith, A.P., Ade, H., Rightor, E.G.: Inner-shell excitation spectroscopy of polymer and monomer isomers of

dimethyl phthalate. J. Phys. Chem. B **101**(13), 2267–2276 (1997). https://doi.org/10.1021/jp963419d

[20] Robin, M.B., Ishii, I., McLaren, R., Hitchcock, A.P.: Fluorination effects on the inner-shell spectra of unsaturated molecules. J. Electron Spectros. Relat. Phenomena **47**, 53–92 (1988). https://doi.org/10.1016/0368-2048(88)85005-9

[21] Horikawa, Y., Tokushima, T., Harada, Y., Takahashi, O., Chainani, A., Senba, Y., Ohashi, H., Hiraya, A., Shin, S.: Identification of valence electronic states of aqueous acetic acid in acid–base equilibrium using site-selective x-ray emission spectroscopy. Phys. Chem. Chem. Phys. **11**, 8676–8679 (2009). https://doi.org/10.1039/B910039C

[22] Tokushima, T., Horikawa, Y., Harada, Y., Takahashi, O., Hiraya, A., Shin, S.: Selective observation of the two oxygen atoms at different sites in the carboxyl group (−COOH) of liquid acetic acid. Phys. Chem. Chem. Phys. **11**, 1679–1682 (2009). https://doi.org/10.1039/B818812B

[23] Prince, K.C., Richter, R., de Simone, M., Alagia, M., Coreno, M.: Near edge x-ray absorption spectra of some small polyatomic molecules. J. Phys. Chem. A **107**(12), 1955–1963 (2003). https://doi.org/10.1021/jp0219045

[24] Golnak, R., Atak, K., Suljoti, E., Hodeck, K.F., Lange, K.M., Soldatov, M.A., Engel, N., Aziz, E.F.: Local electronic structure of aqueous zinc acetate: oxygen k-edge x-ray absorption and emission spectroscopy on micro-jets. Phys. Chem. Chem. Phys. **15**, 8046–8049 (2013). https://doi.org/10.1039/C3CP50686J

[25] Yamamura, R., Suenaga, T., Oura, M., Tokushima, T., Takahashi, O.: ph dependence of aqueous oxalic acid observed by x-ray absorption and emission spectroscopy. Chem. Phys. Lett. **738**, 136895 (2020). https://doi.org/10.1016/j.cplett.2019.136895

[26] Hitchcock, A.P., Brion, C.E.: Inner-shell excitation of formaldehyde, acetaldehyde and acetone studied by electron impact. J. Electron Spectros. Relat. Phenomena **19**(2), 231–250 (1980). https://doi.org/10.1016/0368-2048(80)87006-X

[27] Schreck, S., Pietzsch, A., Kennedy, B., Såthe, C., Miedema, P.S., Techert, S., Strocov, V.N., Schmitt, T., Hennies, F., Rubensson, J.-E., Föhlisch, A.: Ground state potential energy surfaces around selected atoms from resonant inelastic x-ray scattering. Sci. Rep. **6**, 20054 (2016). https://doi.org/10.1038/srep20054

[28] Okajima, T., Teramoto, K., Mitsumoto, R., Oji, H., Yamamoto, Y., Mori,

I., Ishii, H., Ouchi, Y., Seki, K.: Polarized nexafs spectroscopic studies of poly(butylene terephthalate), poly(ethylene terephthalate), and their model compounds. J. Phys. Chem. A **102**(36), 7093–7099 (1998). https://doi.org/10.1021/jp981164t

[29] Zhigunov, D.M., Kamaev, G.N., Kashkarov, P.K., Volodin, V.A.: On raman scattering cross section ratio of crystalline and microcrystalline to amorphous silicon. Appl. Phys. Lett. **113**(2), 023101 (2018). https://doi.org/10.1063/1.5037008
